# An Operando X-ray Absorption Spectroscopy Study on Sensing Characteristics of Vertically Aligned ZnO Thin Film for Methane Gas Sensors

**DOI:** 10.3390/nano12081285

**Published:** 2022-04-09

**Authors:** Suriya Duangmanee, Yingyot Poo-arporn, Pattanaphong Janphuang, Pimchanok Leuasoongnoen, Surangrat Tonlublao, Phitsamai Kamonpha, Natawan Saengchai, Narong Chanlek, Chatree Saisombat, Pinit Kidkhunthod, Rungtiva P. Poo-arporn

**Affiliations:** 1 Biological Engineering Program, Faculty of Engineering, King Mongkut’s University of Technology Thonburi, Bangkok 10140, Thailand; suriyad212@gmail.com; 2 Synchrotron Light Research Institute, Nakhon Ratchasima 30000, Thailand; pattanaphong@slri.or.th (P.J.); pimchanok.envi@gmail.com (P.L.); surangrat@slri.or.th (S.T.); narong@slri.or.th (N.C.); chatree@slri.or.th (C.S.); pinit@slri.or.th (P.K.); 3 School of Physics, Institute of Science, Suranaree University of Technology, Nakhon Ratchasima 30000, Thailand; phitsamai.own1993@gmail.com (P.K.); tawanshai@gmail.com (N.S.)

**Keywords:** ZnO, gas sensor, thin film, methane, operando XAS

## Abstract

In this work, a simple, facile growth approach for a vertically aligned ZnO thin film is fabricated and its application towards methane gas sensors is demonstrated. ZnO thin film was prepared by a combination of hydrothermal and sputtering methods. First, a ZnO seed layer was prepared on the substrate through a sputtering technique, then a ZnO nanorod was fabricated using a hydrothermal method. The surface morphology of the ZnO film was observed by scanning electron microscopy (SEM). A ZnO nanorod coated on the dense seed layer is clearly visible in the SEM image. The average size of the hexagonal-shaped ZnO rod was around 50 nm in diameter, with a thickness of about 1 mm. X-ray absorption near-edge structures (XANES) were recorded to characterize the structural properties of the prepared film. The obtained normalized Zn K-edge XANES of the film showed the characteristic features of ZnO, which agreed well with the standard ZnO sample. The measurement of Zn K-edge XANES was performed simultaneously with the sensing response. The results showed a good correlation between sensor response and ZnO structure under optimal conditions.

## 1. Introduction

Methane (CH_4_) is the most important component of natural gas but is hazardous due to its properties including extreme flammability, promotion of global warming, and its secondary reactions create ground-level ozone. Additionally, the explosive limit for the concentration of CH_4_ gas is 5% [1]. Furthermore, the detection of CH_4_ gas at lower concentrations is essential for preventing its hazards and for warning purposes in leaking gas systems. Nonetheless, detection is extremely difficult due to the chemical inertness of methane, especially at low temperatures [2]. Therefore, it is crucial that highly sensitive sensors for CH_4_ are developed so methane can be detected at low temperatures. As is well known, metal oxides are important materials in catalysis, gas sensors, and energy conversions [3,4].

ZnO is a metal oxide n-type semiconductor which has a direct band gap (Ex = 3.37 eV) with high excitation binding energy (60 meV), exhibiting near UV emission, transparent conductivity, and piezoelectricity. Due to their low cost, high chemical stability, robustness, and versatile preparation methods, they have been widely studied and used for gas-sensing materials. Moreover, the surface states, dimensions, and quantities of adsorbed oxygen all greatly affect gas detection efficiency [5,6,7]. Additionally, the detection response of the ZnO-based gas sensor is significantly affected by operating temperature and gas concentration. Many techniques for the preparation of ZnO nanostructures have been developed, and they are classified as physical and chemical methods such as pulsed laser deposition, magnetron sputtering, electrodeposition, microwave-assisted technique, electron beam evaporation, hydrothermal process, sol-gel methods, chemical vapor deposition, spray pyrolysis, and precipitation [8,9,10,11,12,13]. While the structural and morphological properties of nanomaterials play a vital role in device performance, the design of nanomaterials is a major concern when considering technological applications. Yet the challenge to develop high gas-sensing responses of ZnO nanostructures toward various target gases is still highly desired. 

X-ray absorption spectroscopy (XAS) measurements provide access to specific information about the elements in the electronic structure and geometry of catalyst materials. The two regions of XAS spectra can be divided into two types; the first region provides the X-ray absorption near-edge structure (XANES), while the second region is defined by the modulation of the absorption coefficient up to 1000–1500 eV after the absorption edge, known as the extended X-ray absorption fine structure (EXAFS) region, which probes the local structure around the absorbing atom with a range of up to ∼6 Å. Spectroscopic measurements on real sensors under relevant operating conditions have been applied by X-ray absorption spectroscopy under real “operando” conditions, thereby deriving structure–function relationships helping to explain the interaction of the sensing material and target gas on the surface. Significant advances in synchrotron-based X-ray techniques have provided opportunities to study mechanisms of sensor materials over the last few decades [14,15]. Koziej et al. [16] employ XANES and EXAFS to indicate the presence of fully oxidized Pd in an atomic distribution included in the SnO_2_ lattice. High sensor readings were reported during CO and H_2_ exposure, with no change in the Pd oxidation state. As a result, the data supplied by the operando XAS is useful for improving the understanding of the mechanical elements of gas sensors. The creation of sensing materials benefits greatly from understanding the gas sensing mechanism.

In this work, a gas sensor was fabricated using ZnO nanorods that were prepared through a combinational methodology of the sputtering seed layer and hydrothermal, which showed that nanorod arrays grown with better crystal quality have a vertical substrate orientation. The characteristic features of ZnO are investigated by in situ X-ray absorption spectroscopy (XAS), in which the thin film normalized Zn K-edge XANES were obtained. Moreover, the operando XAS was investigated with a technique to detect the characteristic features of ZnO thin film in real-time with temperature and under methane gas.

## 2. Materials and Methods

### 2.1. Sensor Fabrication

**Figure 1:**
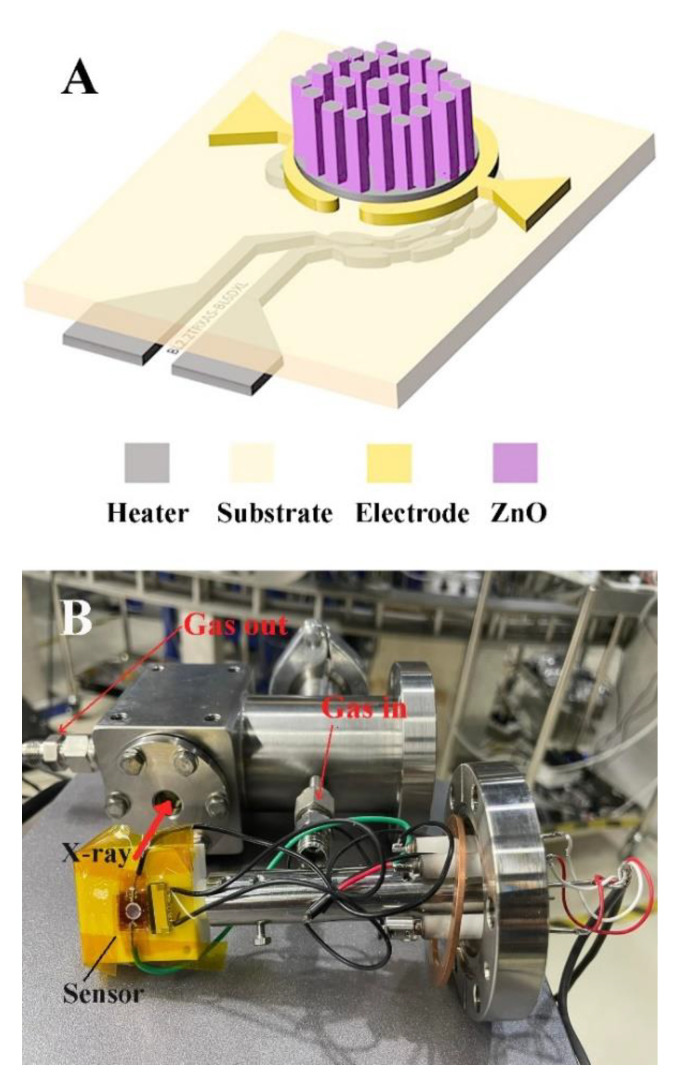
(**A**) Schematic illustration of the ZnO gas sensor and (**B**) the setup of the operando experiment.

The design of the CH_4_ gas sensors presented in this work is shown in Figure 1A, consisting of a sensitive layer based on metal oxide thin films and a pair of gold (Au) annular-like electrodes with a 4.6 mm diameter fabricated on the top side of a polyimide (PI) substrate, while the hot spot made of a platinum (Pt) heater was located on the backside of the PI substrate. The hot spot was designed to localize heating and reduce the input power required to heat the sensor. The hot spot heater was distributed with a wider line section, measuring 300 µm in width and 700 µm in length. The CH_4_ gas sensors were fabricated using a standard cleanroom process at a wafer scale. The hot spot heaters were first patterned on the backside of a 125 µm-thick PI sheet using photolithography. A total of 20 nm-Cr/500 nm-Pt were then deposited using electron beam evaporation, followed by a lift-off process to form the hot spot heater. The next step involved patterning the Au electrode on the top side of the PI sheet by photolithography and deposition of the 20 nm-Cr/150 nm-Au forming electrode of the sensor. Finally, the ZnO thin films, as the sensitive layer, were deposited on the top electrode by a combination of sputtering and hydrothermal processes. The deposition of ZnO thin films was started by RF magnetron sputtering of a ZnO seed layer. Using a glancing-angle deposition (GLAD) technique, the nanorods of ZnO were formed on the surface at an oblique angle. During the sputter deposition process, the vacuum chamber was initially evacuated to a base pressure of 6.0 × 10^−6^ torr. Sputtered targets constructed of 3-inch-diameter and 0.250-inch-thick ZnO (99.9% purity) were used as sputtered targets. The position of the PI substrate and ZnO target tilt angle was designed to be 24°. The ZnO target was set at 70 mm from the centerline of the substrate. A sputtering RF power of 150 W was initially applied to the ZnO target while the chamber was filled with Argon (Ar) to ignite the plasma. The deposition pressure was maintained at 1.4 × 10^−2^ torr and the duration of the deposition pressure was set to 2 h to achieve the ZnO seed layer. The nanorods could be formed on the surface at oblique angles during the ZnO nanorods seed layer deposition process. However, the particles were captured at higher surface points due to the shadowing effect, resulting in the formation of rougher surfaces with columnar structures. Nanorods were fabricated at an oblique angle deposition in the RF magnetron sputtering with an oblique angle deposition procedure, resulting in arrays of nanorods with rough surfaces. Furthermore, a long deposition time was required to grow the thick ZnO nanorods. Instead, a ZnO nanorod was grown on top of the seed layer by a hydrothermal process to complete the sensor fabrication. The hydrothermal process has many advantages, including the ability to grow ZnO nanorods at low temperatures, reduce deposit time for a given thickness, and be less expensive than other deposition techniques such as sputtering and evaporation. The equivalent mole solution of zinc nitrate (Zn(NO_3_)_2_6H_2_O) and hexa-methylenetetramine (C_6_H_12_N_4_, HMTA) was used to hydrothermally synthesize high-density ZnO nanorods. The hydrothermal growth reactions of the ZnO nanorods were conducted at 95 °C for 3 h [17].

### 2.2. Sensor Characterization

The morphology of the prepared film was investigated using a scanning electron microscope (SEM, JEOL JSM-6010LV) at an acceleration voltage of 10–20 kV. X-ray scattering and X-ray absorption spectroscopy (XAS) experiments were conducted to investigate the structural properties of ZnO. Wide-angle X-ray scattering (WAXS) experiments were performed at Thailand’s Synchrotron Light Research Institute (SLRI) beamline 1.1 W. The beam size was 0.3 mm × 0.3 mm while the wavelength was 1.0332 Å. The scattering pattern was recorded with a MAR345 area detector, and the specimen-to-detector distance was approximately 170 mm. The incident angle between the X-ray source and the sample was fixed at 10 degrees. The two-dimensional scattering patterns were calibrated with LaB6 powder and integrated with the GSAS-II software, yielding intensity vs. 2θ in one-dimension scattering patterns. Instrument geometry values such as beam center position, sample to detector distance, and detector tilt angle relative to the x-ray beam were obtained during the calibration process. The 2D patterns’ azimuthal integrations were processed from 25 to 155 degrees azimuth, with 90 degrees being the out-of-plane direction. X-ray absorption near-edge structure (XANES) was performed in the transmission mode at BL2.2: TRXAS of the Synchrotron Light Research Institute, SLRI, Thailand [18]. An energy-dispersive monochromator equipped with a Si (111) single crystal was used. By using an NMOS linear image sensor, Zn K-edge spectra were recorded with an integration time of 1000 ms at an average of 10 scans. The in situ XAS was further performed to study the influence of CH_4_ and temperature on the ZnO film. The prepared film was cut into a circular shape with an outer diameter of 5 mm and then inserted into the test reactor [19]. The Zn K-edge XANES were collected during thermal treatment under 5% *v*/*v* CH_4_/N_2_ at 400 °C with a heating rate of 5 °C/min. The total flow rate of the CH_4_/N_2_ gases was 50 mL/min.

### 2.3. Operando Experiment

To investigate the relationship between the structural properties of the ZnO film and its sensing behavior for CH_4_ gas, in situ X-ray absorption experiments were conducted under 0.5–21.0% *v*/*v* CH_4_/O_2_:N_2_ at 180 °C. The ambient air was represented by the 20% *v*/*v* O_2_ in N_2_ gases. The Zn K-edge XANES spectra of ZnO were rescored simultaneously with signals collected from CH_4_ gas detection. An in situ cell was developed in-house to meet the requirements of the operando experiment (Figure 1B). The test cell was constructed from stainless steel with the dimensions of 50 × 50 × 65 mm^3^. A 10 mm diameter hole was drilled through the cell to allow the X-ray to pass through. Two polyimide windows were placed on either side of the hole to control the inside atmosphere. Inside the cell, the sensor was placed on a Teflon sample holder which was mounted to the CF70 flange. The target CH_4_ concentration was achieved by mass flow controllers (Aalborg) and flowed to the test chamber to investigate sensing performance. The sensor was heated by a built-in heater and controlled by the Keysight e36313a triple output programmable DC power supply with in-house developed software. The CH_4_ sensing-signal was measured by the Keithley 6485 picoammeter. The change in electrical resistance of the sensor was monitored by measuring the current flow in the sensor with a digital picoampmeter at a bias voltage of 5 V. The sampling time was set to 1 s. In operation, the test cell was installed on a three-axis stage, which allows horizontal and vertical alignment of the sensor with the X-ray beam path.

## 3. Results

### 3.1. Morphological Property

**Figure 2:**
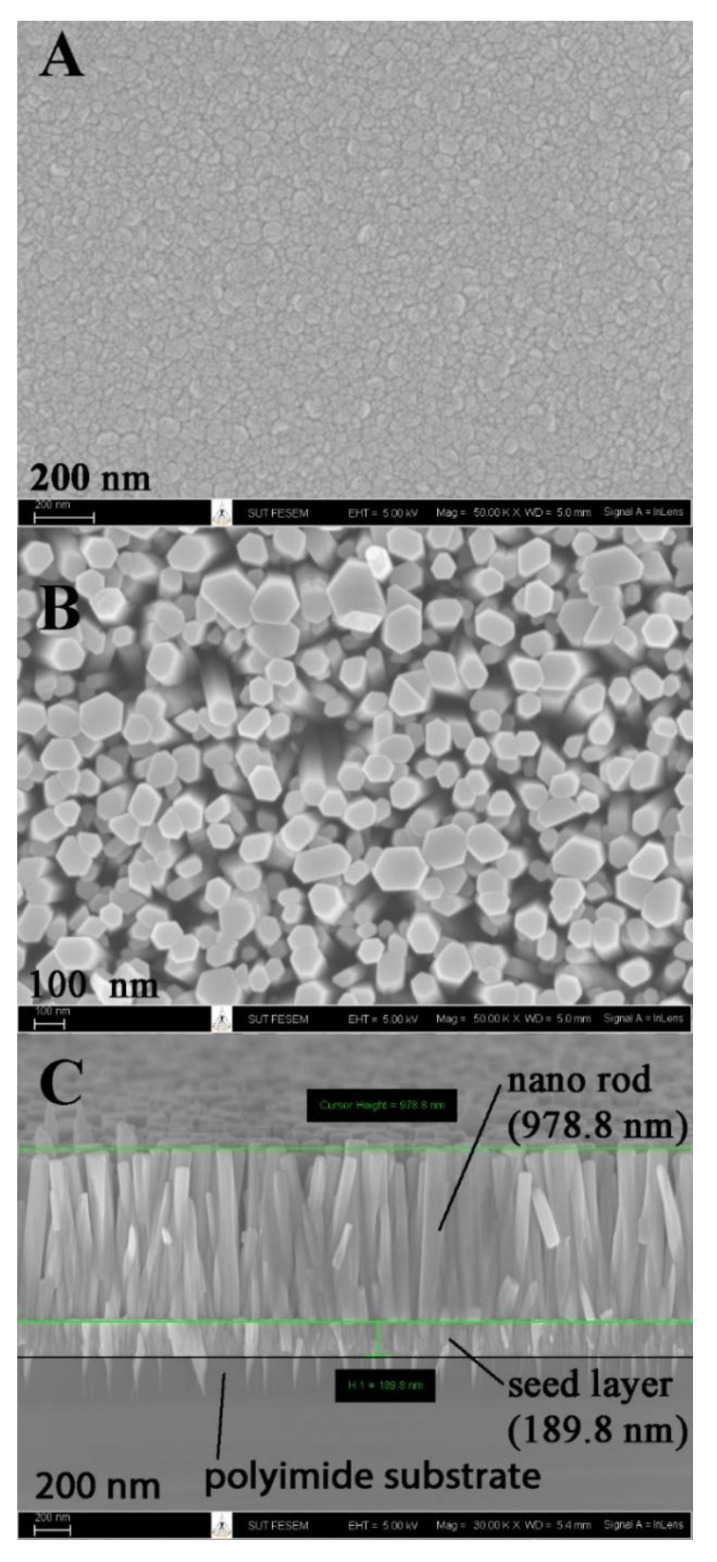
Scanning electron microscopy (SEM) micrographs of the ZnO seed layer prepared by sputtering (**A**) cross-section, (**B**) top view, and (**C**) after growth by the hydrothermal method.

Figure 2 presents the SEM images of the ZnO active layer of the CH_4_ gas sensor prepared by the combination of sputtering and hydrothermal methods. To grow the vertically aligned ZnO nanorod, prior fabrication of the ZnO seed layer onto the substrate was necessary [20]. Thus, in this work, the sputtering technique was employed to fabricate the ZnO seed layer onto the polyimide substrate. The evolution of the surface morphology of ZnO films is shown in Figure 2A. The grain boundaries of seed particles were highly coalesced with a distinct close-packed structure. The shape of seed particles was irregular, with sizes ranging from 30 to 100 nm. The size of the seed particle directly affects the stress and surface-to-volume ratio of the film. The size increase tends to reduce the values of both parameters. Furthermore, the larger grain size normally exhibits better electrical properties of the film.

Figure 2B,C present the top view and cross-section of the ZnO nanorods grown on the ZnO seed layer by the hydrothermal method. The nanorods had an irregular hexagonal form, with rod sizes ranging from 50 to 150 nm. The seed layer and nanorods were 189.8 nm and 978.8 nm thick, respectively, as estimated from the cross-section SEM image. The nanorod density was estimated to be around 13 million per mm^2^, with an aspect ratio of approximately 10.0. The reaction mechanisms of ZnO nucleation and crystal growth are as follows [21]:

C_6_H_12_N_4_ + 6H_2_O   →   6HCHO + 4NH_3_ (1)

NH_3_ + H_2_O   →   NH_4_^+^ + OH^−^ (2)

Zn^2+^ + 2OH^−^   →   Zn(OH)_2_ (3)

Zn(OH)_2_   →   ZnO + H_2_O (4)

The self-assembling of nanorods on seed particles requires anisotropic crystal growth. Pacholski et al. [22] reported that ZnO nanoparticles are favorable for orientated attachment because crystalline fusion of the attached particles leads to a gain in lattice-free energy and free energy of polycondensation. Moreover, during the hydrothermal process, the non-polar surfaces of the wurtzite structure of the ZnO nanorods can be attached by HMT which subsequently inhibits lateral growth along the a-axis of the nanorods [23].

Thus, the crystal growth of ZnO nanorods was favored on the c-axis (002 plane). Interestingly, a tip-like structure of sputtered ZnO was observed underneath the seed layer. Penetration of ZnO into the polyimide improves the adhesion strength of the ZnO film to the substrate.

### 3.2. Structural Property 

**Figure 3:**
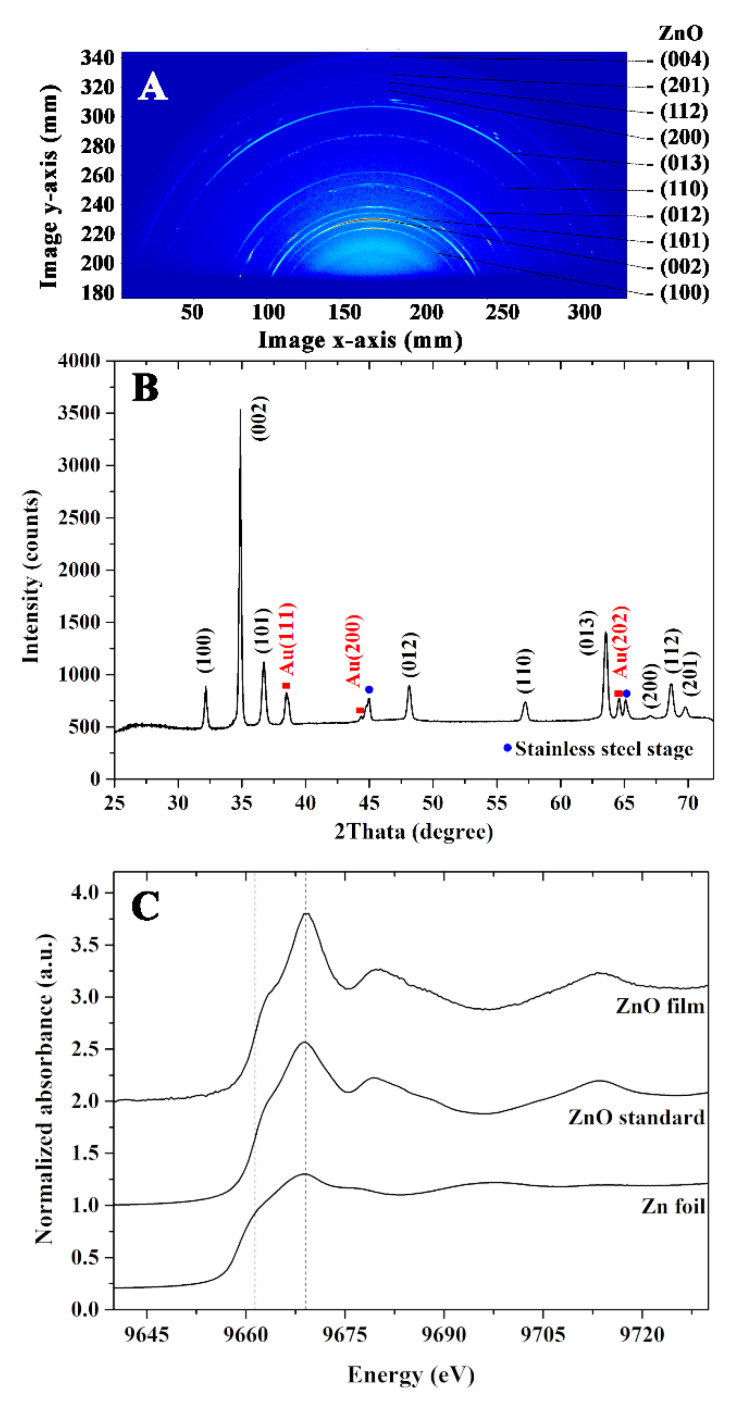
(**A**) WAXS patterns, (**B**) X-ray diffraction pattern, and (**C**) normalized Zn K-edge XANES of the ZnO nanorods thin film.

The WAXS pattern represents a specific orientation of ZnO, Au electrode, and stainless-steel stage. The relative intensity of each orientation is shown in Figure 3A. The one-dimensional scattering intensity profiles as shown in Figure 3B were integrated radially from 25 to 75 degrees of the two-dimensional scattering patterns, where 90 degrees was an out-of-plane direction. The scattering pattern was indexed by Software Match using the combined-package FullProf Software based on the PDF2 Database. The ZnO (100) and (002) components of the hexagonal ZnO nanorod were directed perpendicular to the substrate. The strong peak intensity of the diffraction pattern on the (002) plane confirmed the crystal growth of ZnO nanorods along the c-axis. Other scattering features, such as ZnO (012) and ZnO (101), are combinations of the in-plane and out-of-plane components. The peak splitting of ZnO (002) in the one-dimension scattering intensity profile could be derived from a slightly different lattice constant. 

In addition to the XRD analysis, an XAS measurement was further performed to investigate the structural properties of the prepared film. To study the photo-absorption characteristics of the Zn atom, XANES spectra were recorded at the energy range of 9640–9730 eV. The normalized Zn K-edge XANES of Zn foil, standard ZnO, and ZnO thin film are shown in Figure 3C. When an X-ray was exposed to the Zn atom with sufficient energy, the core electron was ejected and produced the photoelectron that subsequently created the characteristic XANES spectrum. The comparison of Zn K-edge XANES revealed the similarity of edge energy and absorption features obtained from the standard ZnO and the film. These findings confirm the successful preparation of the ZnO thin film by a combination of the sputtering and hydrothermal methods. 

### 3.3. Sensing Ability 

**Figure 4:**
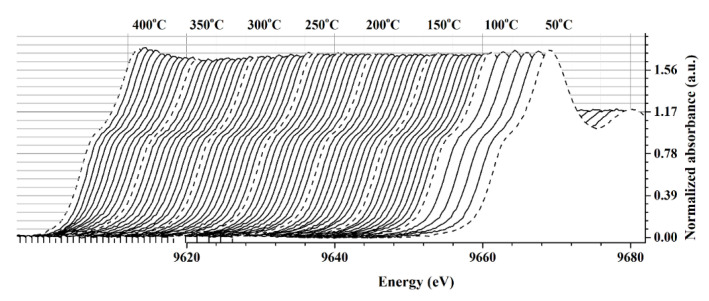
The normalized Zn K-edge XANES of ZnO film under the CH_4_ atmosphere and applied temperature.

To study the interaction between ZnO film and target gas, the in situ XANES experiment under CH_4_ atmosphere and applied temperature was performed. The Zn K-edge XANES of ZnO film were recorded at 10 °C intervals from 50 °C to 100 °C and at 5 °C intervals from 105 °C to 400 °C (Figure 4). Although the XAS technique is typically used to determine the bulk property of a sample, it can also be used to determine the surface property of a sample in some cases. The interaction between ZnO film and CH_4_ gas was determined using the changing white line intensity in the Zn K-edge XANES spectrum for our sample. At temperatures lower than 250 °C, there were no significant changes in white line peak intensity. The white line is attributed to the primary sharp rise in the XANES spectrum and corresponds to transitions of electrons to empty certain states slightly below the continuum of free electron states. As a result, the intensity of the white line is strongly related to the density of the Zn atom in its empty state. In the presence of CH_4_ gas, CH_4_ molecules chemisorb on the surface oxygen of the ZnO film. The donation of an electron to the ZnO system reduced the empty state of Zn atoms (Equations (5) and (6)), lowering the white line intensity of the Zn K-edge XANES spectrum. The intensity of the white line began to reduce at 275 °C and exhibited a drastic change at temperatures between 300 °C and 350 °C. When the temperature was increased to 400 °C, however, the regeneration of white line intensity could be observed. It should be noted that the maximum temperature for the in situ XANES experiment was limited by the degradation temperature of the polyimide substrate.

ZnO is a d^10^ n-type semiconductor with a filled valence band that is primarily oxygen 2p [24]. To detect the CH_4_ gas, its sensing mechanism involves the chemisorption of atmospheric oxygen and the removal of chemisorbed oxygen by reducing the gas as follows [25]:

O_2_ + 2e^−^   →   2O^−^_ads_ (5)

R + O^−^_ads_   →   RO_ads_ + e^−^ (6)

where, R is the reducing gas. Under oxygen adsorption, the trapping of electrons from the bulk of material causes an electronic change in the oxide surface. At moderate temperatures, the presence of a reducing gas could remove the chemisorbed oxygen from ZnO. These two irreversible reactions proceed in opposite directions and would reach a steady state at a given reducing gas concentration. Since the presence of a white line strongly depends on the electron density of the unoccupied state, for ZnO film, the reduction in its intensity under the CH_4_ atmosphere was correlated with the removal of chemisorbed oxygen atoms from its surface. As a result, it is plausible that the optimal temperature for detecting CH_4_ gas with this ZnO film was around 300–350 °C.

**Figure 5:**
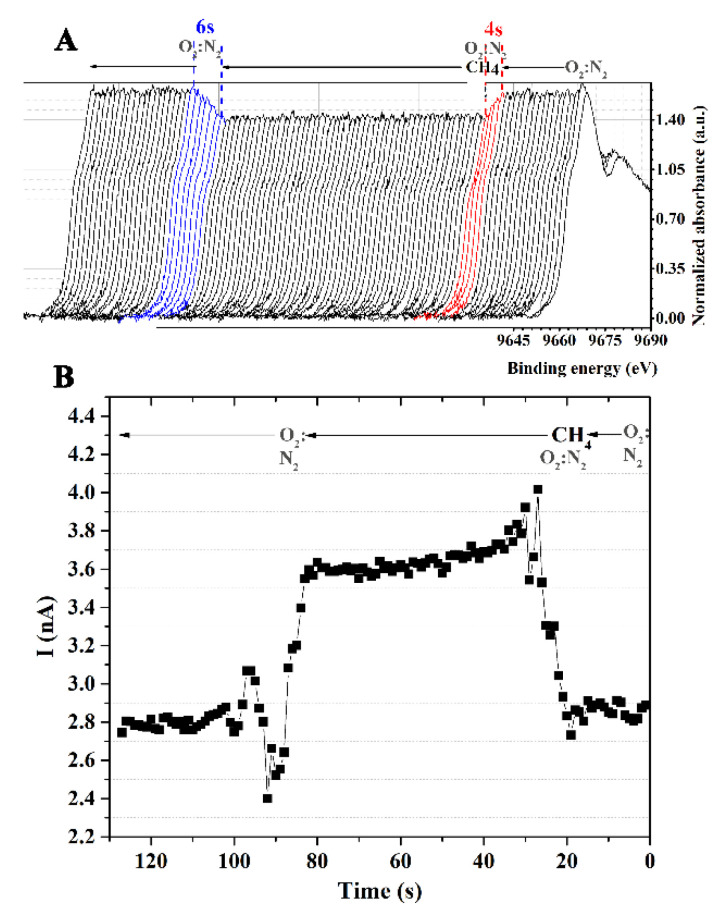
(**A**) The normalized Zn K-edge XANES of ZnO film under the CH_4_ atmosphere and at 180 °C and (**B**) the current vs. time.

The operando XANES experiment was performed to study the correlation between the sensing response and structural properties of the ZnO film. The Zn K-edge XANES was recorded simultaneously with the measurement of the sensor signal under a 5% *v*/*v* CH_4_ atmosphere at 180 °C. A data acquisition time of 1000 ms was used to record XANES. As discussed previously, because of the increased electron density in the unoccupied state, the white-line intensity of ZnO could be reduced in a CH_4_ environment. It is reported in the literature that gas sensing with semiconductor metal oxide devices comprises two crucial actions: receptor and transducer capabilities [26]. The absorption of reducing gas by the ZnO sensor reduced its potential barrier, allowing electrons to flow freely and lowering the electrical resistance that produced the signal. Hence, the ZnO sensor response signal was directly correlated to the white line intensity. As seen in Figure 5A, before CH_4_ exposure, the intensity of the white line peak remained unchanged under the O_2_:N_2_ environment, confirming the sensor’s undetected signal. When CH_4_ occurred, the white line’s intensity decreased over time, while the detecting signal significantly increased. Both values had reached their typical levels after around 4 s of exposure time. These numbers could be indicative of the sensor’s response times. To retreat the ZnO surface, O_2_:N_2_ gas was fed into the test chamber instead of CH_4_/O_2_:N_2_ mixture. The results showed that the sensor surface could be recovered in approximately 6 s. 

**Figure 6:**
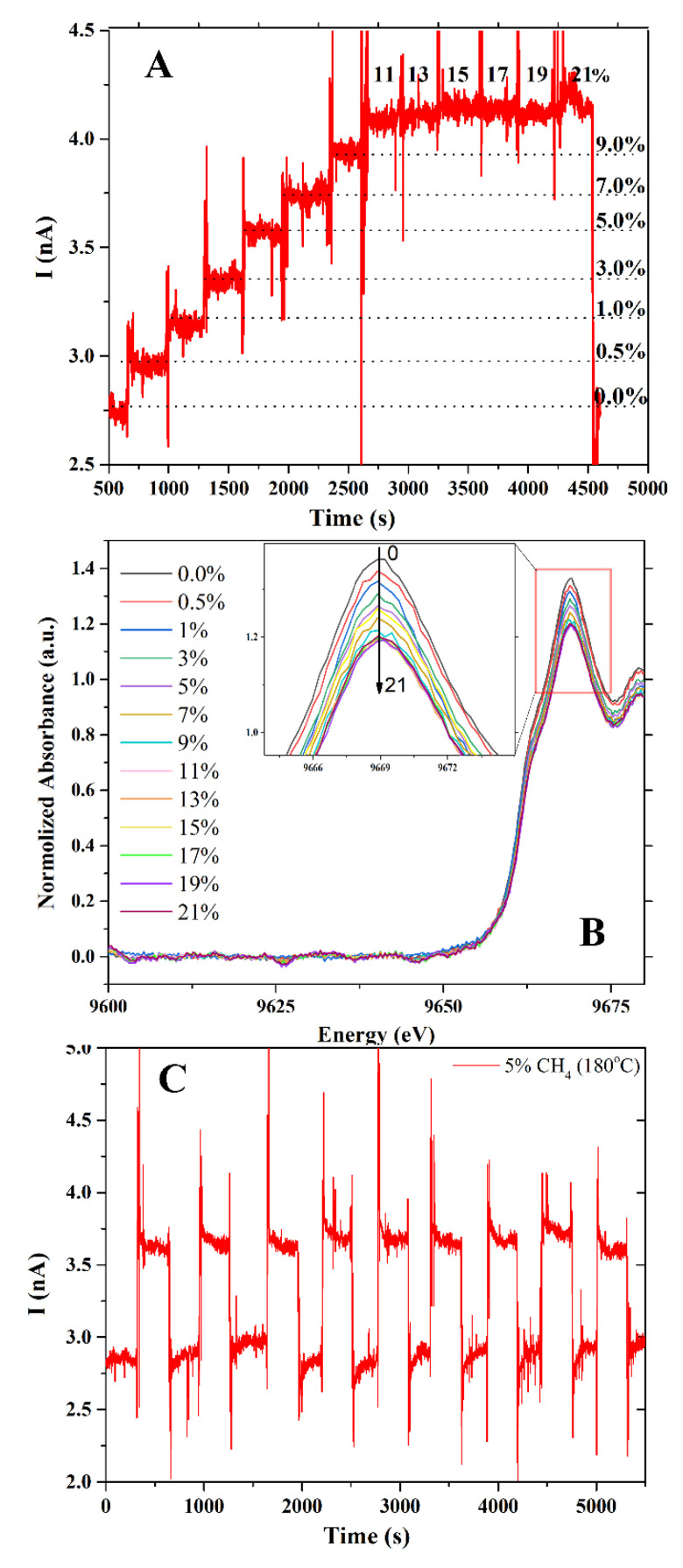
(**A**) Sensor signal under CH_4_ gas at various concentrations, (**B**) the normalized Zn K-edge XANES of ZnO film and (**C**) nine consecutive exposures.

At 180 °C, Figure 6A depicts the sensor signal of a ZnO sensor exposed to 0, 0.5, 1, 3, 5, 7, 9, 11, 13, 15, 17, 19 and 21% *v*/*v* CH_4_/O_2_:N_2_ gas. The sensor response is shown to be dependent on the gas concentration. A current of roughly 3 nA was recorded under 0.5% *v*/*v* CH_4_/O_2_:N_2_ gas. The sensor current was increased to 4.2 nA when the CH_4_ concentration was increased to 11% *v*/*v*. No indication of signal increase was observed when the sensor was exposed to higher CH_4_ concentrations. The amount of CH_4_ adsorbed on the sensor surface is related to this behavior. The higher the ethylene content, the greater the response. These results agreed well with the Zn K-edge XANES spectra presented in Figure 6B. In contrast to the sensor signal, the white line intensity of ZnO decreased when CH_4_ concentration increased. At CH_4_ concentrations higher than 11% *v*/*v*, the white line intensity remained unchanged. These results confirmed the saturation of CH_4_ gas on the ZnO surface. To study the stability and repeatability of the sensor, it was exposed to CH_4_ gas for nine consecutive exposures (Figure 6C). The sensors have an excellent response and recovery pattern, implying that the sensor is stable and reproducible after multiple exposures.

## 4. Conclusions

The ZnO thin film was fabricated on the polyimide substrate using a combinational method of sputtering and hydrothermal technique. As evidenced by cross-section SEM, the vertically aligned ZnO nanorods were grown on the seed layer. The tip-like structure underneath the seed layer played an important role in improving the adhesion ability of the ZnO film on the substrate. The in situ Zn K-edge XANES measurement could be applied to examine the optimal operating temperature. The operando experiment was successfully performed to determine the correlation between structural property and sensing ability of ZnO thin film as the CH_4_ sensor.

## Data Availability

The data is available on reasonable request from the corresponding author.
